# Egg‐shaped aortic thrombi associated with COVID‐19

**DOI:** 10.1002/jgf2.491

**Published:** 2021-08-22

**Authors:** Ibuki Kurihara, Takahiko Fukuchi, Hanako Yoshihara, Kenichi Sakakura, Hitoshi Sugawara

**Affiliations:** ^1^ Division of General Medicine Department of Comprehensive Medicine Saitama Medical Center Jichi Medical University Saitama City Japan; ^2^ Division of Cardiovascular Medicine Department of Comprehensive Medicine Saitama Medical Center Jichi Medical University Saitama City Japan

**Keywords:** cardiovascular medicine, infectious diseases, internal medicine, respiratory disease, venous thrombosis

## Abstract

We experienced a case with multiple arterial and venous thromboses associated with COVID‐19. During this pandemic, physicians should consider COVID‐19 in patients with unexplained thrombosis.

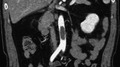

A 52‐year‐old Japanese man who was a current smoker and had a medical history of dyslipidemia presented with fever, a 14‐day history of malaise, and a sore right foot for 2 days. He had no medical history of any thrombosis‐predisposing illnesses such as stroke, myocardial infarction, superior mesenteric arterial occlusion, or peripheral arterial occlusion. On examination, the patient had hypoxemia, with an SpO_2_ level of 83% on ambient air, peripheral cyanotic discoloration of the right foot, and unpalpable right popliteal and dorsal arteries. Laboratory tests revealed a white blood cell count of 9510/μl, hemoglobin of 9.3 g/dl, platelet count of 471,000/μl, aspartate aminotransferase of 47 U/L, alanine aminotransferase of 26 U/L, lactate dehydrogenase of 551 U/L, creatine kinase of 634 U/L, creatinine of 0.7 mg/dl, HbA1c of 6.3%, C‐reactive protein of 9.33 mg/dl, prothrombin time activity of 83.7%, activated partial thromboplastin time of 27.8 s, fibrinogen of 564 mg/dl, D‐dimer of 10.9 μg/ml, and negativity for protein C, protein S, and antiphospholipid antibodies (anticardiolipin immunoglobulins M and G, and anti‐β2‐glycoprotein I). Enhanced computed tomography (CT) revealed bilateral pulmonary crazy paving (Figure 1), a 4 × 1.5 × 1.5‐cm egg‐shaped thrombus in the abdominal aorta (Figure 2), and thrombi in the right pulmonary, left popliteal, bilateral peroneal, and right posterior tibial arteries, but no left atrial emboli, aortic aneurysm, dissection, or deep vein thrombosis. COVID‐19 was confirmed by reverse‐transcriptase polymerase chain reaction test for SARS‐CoV‐2 on admission. We diagnosed the patient with multiple arterial and venous thromboses associated with COVID‐19. He was administered intravenous heparin with an activated partial thromboplastin time of 51–85 s,^1^ followed by apixaban, resulting in symptom amelioration. On hospital day 9, reexamined enhanced CT revealed a 2.8 × 1.0 × 0.8 cm diminished thrombus in the abdominal aorta but no new thromboses in other arteries. He was discharged on hospital day 18. The patient provided written informed consent for publication.

**FIGURE 1:**
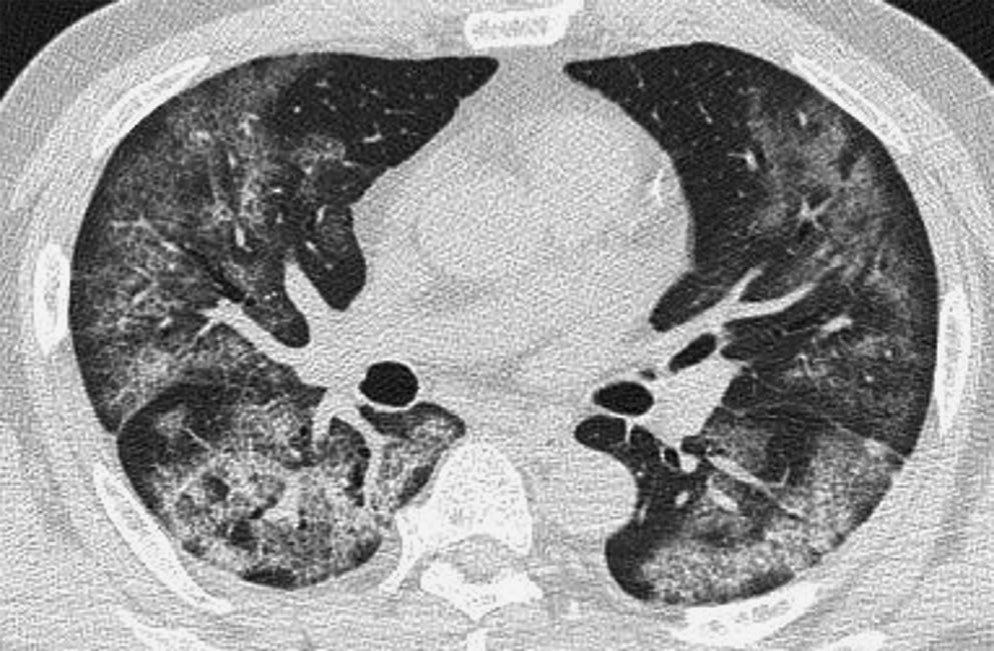
Chest computed tomography showing bilateral pulmonary crazy paving appearances

**FIGURE 2:**
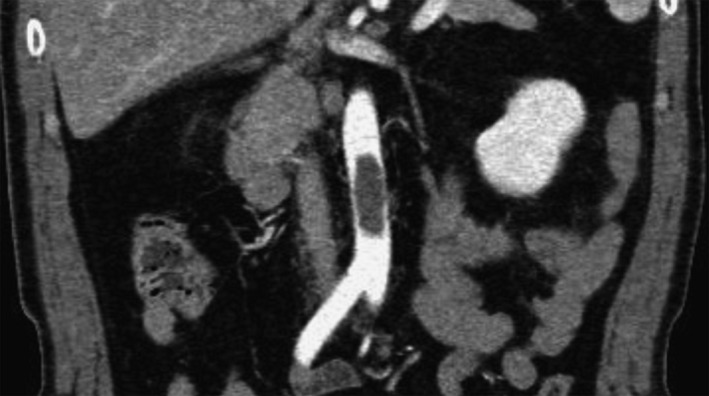
Enhanced computed tomography showing a 4 × 1.5 × 1.5 cm egg‐shaped thrombus in the abdominal aorta

Aortic thrombus is uncommon, even in common hypercoagulable states such as sepsis, polycythemia, disseminated intravascular coagulation, pregnancy, malignancy, antiphospholipid syndrome, and protein S deficiency.^2^ Approximately 8%–65% of aortic emboli originate from the left atrium, and primary thrombus accounts for the remaining 35%–92%. Most cases featured underlying atherosclerosis, aneurysm, or dissection,^3^ but not our case. A literature search on PubMed retrieved 20 cases in 14 case reports on aortic thrombosis associated with COVID‐19, of which 8 (40%) cases had aortic round thrombi similar to our patient (Table S1). COVID‐19 may predispose patients to thrombosis in both arterial and venous circulations.^4^ SARS‐CoV‐2 directly infects endothelial cells, inducing apoptosis and endothelial inflammation, which can result in widespread endothelial dysfunction and a procoagulant state.^5^ Furthermore, hypoxia caused by pneumonia, acute respiratory distress, or pulmonary thrombus may lead to a hypercoagulable state because hypoxia‐inducible transcription factors can directly activate platelets and coagulation factors.^6^ COVID‐19 can cause thrombotic events and aortic thrombi, but these events could be underestimated because of the asymptomatic state and delayed enhanced CT owing to infection control in the hospital. Physicians should consider COVID‐19 as an underlying condition in patients with unexplained thrombosis.

## CONFLICT OF INTEREST

The authors have stated explicitly that there are no conflicts of interest in connection with this article.

## Supporting information

Table S1

## References

[1] Basu D , Gallus A , Hirsh J , Cade J . A prospective study of the value of monitoring heparin treatment with the activated partial thromboplastin time. N Engl J Med. 1972;287(7):324–7. 10.1056/NEJM197208172870703 5041701

[2] Aksu AO , Demirkazik FB . Floating aortic thrombus in a patient with non‐Hodgkin’s lymphoma. Diagn Interv Radiol. 2010;16(1):63–5. 10.4261/1305-3825.DIR.1226-07.1 19813176

[3] Babu SC , Shah PM , Nitahara J . Acute aortic occlusion ‐ factors that influence outcome. J Vasc Surg. 1995;21(4):567–75. 7707562 10.1016/s0741-5214(95)70188-5

[4] Bikdeli B , Madhavan MV , Jimenez D , Chuich T , Dreyfus I , Driggin E , *et al* COVID‐19 and thrombotic or thromboembolic disease: implications for prevention, antithrombotic therapy, and follow‐up: JACC State‐of‐the‐Art Review. J Am Coll Cardiol. 2020;75(23):2950–73. 10.1016/j.jacc.2020.04.031 32311448 PMC7164881

[5] Varga Z , Flammer AJ , Steiger P , Haberecker M , Andermatt R , Zinkernagel AS , *et al* Endothelial cell infection and endotheliitis in COVID‐19. Lancet. 2020;395(10234):1417–8. 10.1016/S0140-6736(20)30937-5 32325026 PMC7172722

[6] Frantzeskaki F , Armaganidis A , Orfanos SE . Immunothrombosis in acute respiratory distress syndrome: cross talks between inflammation and coagulation. Respiration. 2017;93(3):212–25. 10.1159/000453002 27997925

