# Supplementary material for: Egg‐shaped aortic thrombi associated with COVID‐19
Source: J Gen Fam Med. 2021 Aug 22;23(2):116–7. doi: 10.1002/jgf2.491 (PMC8657516; doi:10.1002/jgf2.491)
Supplement: Supplementary file 1 — Table S1 [file JGF2-23-116-s001.docx]

**Supplementary Table 1.** Previously published case reports on aortic thrombosis associated with COVID-19.

| Author | Reference | Description of thrombosis |
| --- | --- | --- |
| Kashi M et al. | Thromb Res. 2020 Aug; 192: 75-7. | No images |
| Le Berre A et al. | Diagn Interv Imaging. 2020 May; 101(5): 321-322. | Floating and not round thrombus |
| Gandotra P et al. | J Emerg Med. 2021 Feb; 60(2): 223-225. | Round thrombus |
| Gomez-Arbelaez D et al. | Ann Vasc Surg. 2020 Aug; 67: 10-13. | Floating and round thrombus in two of four cases |
| Mosbahi S et al. | Eur J Cardiothorac Surg. 2020 Oct; 58(4): 870. | Floating but not round thrombus in the aortic arch |
| de Carranza M et al. | J Thromb Thrombolysis. 2021 Jan;51(1):237-242. | Three cases of floating and round thrombi |
| Mukherjee A et al. | Am J Trop Med Hyg. 2020 Nov;103(5):1989-1992. | Floating and round thrombus |
| Roncati L et al. | Ann Vasc Surg. 2020 Aug; 67: 8-9. | Triangular thrombus |
| Azouz E et al. | Intensive Care Med. 2020 Jul; 46(7): 1464-1465. | Floating and not round thrombus |
| Mullan C et al. | J Thorac Cardiovasc Surg. 2020 Aug; 160(2): e13-e14. | Floating and not round thrombus |
| Katchanov J et al. | Ann Emerg Med. 2020 Sep; 76(3): 373-374. | Extensive thromboembolic material, particularly in the center of the abdominal aortic lumen |
| Silingardi R et al. | J Vasc Surg. 2020 Sep; 72(3): 1152-1153. | Floating and not round thrombus |
| Vulliamy P et al. | Br J Haematol. 2020 Jun; 189(6): 1053-1054. | Floating and not round thrombus |
| Vacirca A et al. | Int J Infect Dis. 2020 Jul; 96: 590-592. | Floating and round thrombus |
